# Research progress on using biological cathodes in microbial fuel cells for the treatment of wastewater containing heavy metals

**DOI:** 10.3389/fmicb.2023.1270431

**Published:** 2023-09-18

**Authors:** Hui Wang, Pengxiang Zhai, Xizi Long, Jianghang Ma, Yu Li, Bo Liu, Zhiqiang Xu

**Affiliations:** ^1^State Key Laboratory of Eco-Hydraulics in Northwest Arid Region, Xi'an University of Technology, Xi'an, China; ^2^Department of Municipal and Environmental Engineering, School of Water Resources and Hydro-Electric Engineering, Xi'an University of Technology, Xi'an, China; ^3^Key Laboratory of Typical Environmental Pollution and Health Hazards of Hunan Province, School of Public Health, Hengyang Medical School, University of South China, Hengyang, China

**Keywords:** microbial fuel cell, biocathode, heavy metal wastewater, cathodic reduction, electron transfer

## Abstract

Various types of electroactive microorganisms can be enriched to form biocathodes that reduce charge-transfer resistance, thereby accelerating electron transfer to heavy metal ions with high redox potentials in microbial fuel cells. Microorganisms acting as biocatalysts on a biocathode can reduce the energy required for heavy metal reduction, thereby enabling the biocathode to achieve a lower reduction onset potential. Thus, when such heavy metals replace oxygen as the electron acceptor, the valence state and morphology of the heavy metals change under the reduction effect of the biocathode, realizing the high-efficiency treatment of heavy metal wastewater. This study reviews the mechanisms, primary influencing factors (e.g., electrode material, initial concentration of heavy metals, pH, and electrode potential), and characteristics of the microbial community of biocathodes and discusses the electron distribution and competition between microbial electrodes and heavy metals (electron acceptors) in biocathodes. Biocathodes reduce the electrochemical overpotential in heavy metal reduction, permitting more electrons to be used. Our study will advance the scientific understanding of the electron transport mechanism of biocathodes and provide theoretical support for the use of biocathodes to purify heavy metal wastewater.

## Highlights

- Systematic investigation of the electron-transfer mechanism of a biocathode.
- Discussion of the key factors influencing heavy metal removal by a biocathode.
- Summary of the microbial community structure on a biocathode.

## 1. Introduction

Heavy metal pollution is a severe water problem globally (Nan et al., 2013). Untreated heavy metal effluents can cause serious water and soil pollution in surrounding areas, resulting in potentially significant harm to humans (Kapahi and Sachdeva, 2019). Traditional remediation methods, such as chemical precipitation, ion exchange, and membrane filtration, can reduce the biological effectiveness of heavy metals in the environment by converting them to an inactive state (Azimi et al., 2017; Zamri et al., 2017). However, these techniques are limited by the treatment environment and can cause secondary contamination and incur high costs. Recently, researchers have applied bioelectrochemical systems based on extracellular electron transfer from microorganisms, such as microbial fuel cells (MFCs), to the remediation of heavy metals in wastewater. In a typical MFC system, electroactive microorganisms metabolize and oxidize organic matter under anaerobic conditions to produce electrons and protons. The electrons reach the cathode from the external circuit, while the protons reach the cathode through the proton exchange membrane. Here, the electrons, protons, and final electron acceptor (typically oxygen) that reach the cathode undergo a reduction reaction in the cathode chamber, producing H_2_O and generating electricity (Santoro et al., 2017).

Certain heavy metals with high redox potentials, such as V(V), Cr(VI), and Cu(II), can replace oxygen as the electron acceptor in MFCs and obtain electrons from the cathode, reducing the toxicity of heavy metals via chemical reduction or producing easily recoverable monomers (Wu et al., 2015; Qiu et al., 2017; Wang et al., 2020). However, cathode activation energy and ohmic losses, as well as mass-transfer processes, reduce the performance of the cathode (Rismani-Yazdi et al., 2008). Therefore, the kinetic performance of the MFC can be improved by increasing the reaction area or oxidant concentration, lowering the activation potential, and reducing activation losses (Massazza et al., 2021). Regarding ohmic losses, reducing the internal resistance of the electrode and electrolyte drives the electron-and proton-transfer processes and improves the power-generation performance of the MFC (Lawson et al., 2020). Mass-transfer loss, owing to reactant depletion or product accumulation, typically occurs at high current densities (Choi and Sang, 2016). Hence, modifying the cathode materials, increasing the ionic strength and oxygen concentration, and reducing the reduction reaction overpotential of oxygen at the cathode can reduce the internal resistance to cathodic mass transfer and improve the cathode reaction rate (Venkata Mohan et al., 2014). This ultimately results in an improvement in cathode performance. When microorganisms are enriched on the cathode, thereby forming a biocathode, electroactive microorganisms can significantly reduce the charge-transfer resistance, accelerate electron transfer, and effectively transfer electrons from the cathode to heavy metal ions with a high valence state. Moreover, the interaction between the microorganisms and electrode surface can increase the initial potential of the biocathode and reduce the energy required for heavy metal reduction. Therefore, microorganisms can act as catalysts to obtain electrons directly or indirectly from the cathode and transfer them to electron acceptors, such as oxygen and heavy metals, promoting their reactions on the biocathode (Wu et al., 2015). This study systematically investigated the electron-transfer mechanism of biocathodes, key factors influencing heavy metal removal by biocathodes (e.g., electrode material, initial concentration and species of heavy metals, pH, and electrode potential), and influence of the microbial community structure on the electrical production performance and removal effect of MFCs. The results of this study will provide new ideas and important references for using biocathode MFCs to treat heavy metal wastewater.

## 2. Mechanism of electron transfer in the biocathode

**Figure 1:**
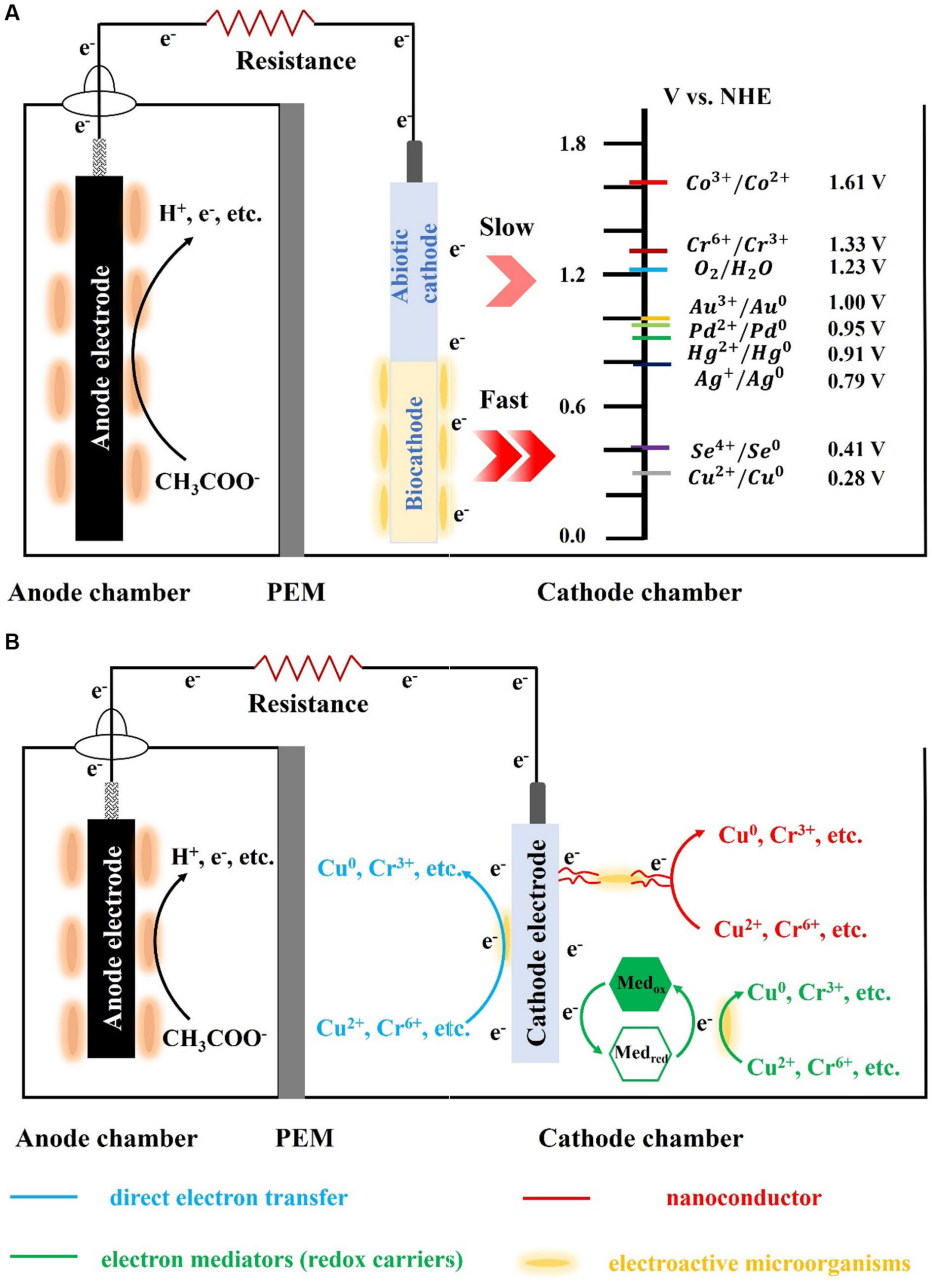
Schematic of the biocathode-mediated heavy metals reduction (**A**: the abiotic-and bio-cathode; **B**: heavy metal reduction via different pathway).

Electron transfer from the cathode to microorganisms involves both direct and indirect electron transfer (Figure 1). Direct electron transfer is the process in which the cathode comes into direct contact with cellular structures, such as the nanowire, membrane proteins, and cell wall, and acquires electrons. Microorganisms that perform direct electron transfer typically have transmembrane structures, such as pore porin-cytochrome complexes (e.g., MtrCAB, MtrDEF, and OmabcB), and polyferric heme-assembled nanoconductors (e.g., OmcZ, OmcS, and OmcE) in their outer membrane (Breuer et al., 2015; Prathiba et al., 2022). For example, the representative anisotropic metal-reducing bacterium *Shewanella* sp. can rapidly reduce reduced fumarate via transmembrane complexes that can channel electrons from the outer membrane to periplasmic fumarate reductase (Li et al., 2014). The transmembrane complex can also combine with the riboflavin/flavin mononucleotide and other autocrine substances to form flavoproteins, which can change electron transfer from a two-electron process to one that involves a single electron, thereby significantly increasing the current of the biocathode (Tokunou et al., 2016). *Shewanella* sp. and *Geobacter* sp. lack the ability to fix carbon or utilize cathodic electron proliferation to colonize the electrode surface indefinitely; therefore, researchers are interested in biocathodes made of autotrophic microorganisms. *Sideroxydans lithotrophicu*, an iron-oxidizing bacterium, oxidizes Fe(II)-smectite to obtain electrons via the heme protein CyC2 with MotA (Zhou et al., 2022). *Rhodopseudomonas palustris* strain TIE-1, an iron-and photo-oxidizing bacterium, obtains electrons from iron oxides, divalent iron, and electrodes (Bose et al., 2014). Researchers deleted the transmembrane complex pioABC [PioA (periplasmic decahemoglobin cytochrome), PioB (outer membrane pore protein), and PioC (periplasmic high-potential iron–sulfur cluster protein)] to obtain *ΔpioABC* photobiofilms. These photobiofilms received 30% less current than the wild type strain, and the mutant photobiofilms were approximately 8–10 times less dense than the wild type. In addition to iron-oxidizing bacteria that can use heme to directly uptake electrons, another naturally widespread class of sulfate-reducing bacteria can also form biocathodes. Deng et al. found that *Desulfovibrio ferrophilus* IS5 can actively express outer membrane heme to maintain its energy requirements by directly obtaining electrons from the environment when the availability of external organic carbon is insufficient (Deng et al., 2018). Recent studies have shown that IS5 can express heme directly from the outside to meet its energy needs and use nanoconductors to obtain electrons (Liang et al., 2021). However, most microorganisms do not possess electron transfer capabilities, owing to the presence of lipopolysaccharide and peptidoglycan in their outer membrane. Therefore, they require exogenous or autocrine electron mediators (i.e., redox carriers) to achieve electron transfer between electrodes and microorganisms, which is considered an indirect electron-transfer process. For example, owing to its small dissociation constant, *Shewanella oneidensis* MR-1 uses autocrine riboflavin, which readily acts as an electron mediator to transfer electrons to Cr(VI) and remove them via reduction (Pang et al., 2013). Recently, the exogenous presence of Fe(II) significantly increased the ability of MR-1 to accept electrons from the cathode (Abuyen and El-Naggar, 2023). These mediators can significantly reduce the internal resistance to charge transfer and internal diffusion resistance of the cathode, increasing the electron-transfer rate. They can also reduce the cathodic reduction overpotential by obtaining electrons from the cathode and transferring them to the electron acceptor. Electron uptake by microorganisms at a biological cathode is primarily accomplished by direct electron transfer. However, cathode microorganisms with denitrification and chemical synthesis functions are often difficult to cultivate and isolate, and the identification of electron-transfer pathways is also difficult, which greatly limits their practicality.

## 3. Key factors affecting the removal of heavy metals using biocathodes

### 3.1. Electrode materials

The electrode acts as a carrier for microorganisms and exchange site for electrons. The surface properties of the electrode material, such as surface roughness, biocompatibility, and electron-transfer rate between microorganisms and the electrode surface, can affect the biocatalytic activity of the biocathode. Carbonaceous materials (e.g., carbon rods, carbon mats, carbon fibers, and graphite) have good electrical conductivity and physical and chemical stabilities; thus, they are more widely used as biocathode materials for the MFC treatment of heavy metal wastewater. Liu et al. (2021) constructed a biocathode using stainless steel, which enhanced the migration of Cu(II) to the cathode in a single-chamber sediment MFC and formed Cu_2_O and CuO in the cathode. The removal rate of Cu(II) in the sediment attained 57%, which was 1.7 times higher than that of the non-biocathode MFC. Fei et al. (2017) constructed a three-dimensional biocathode by loading carbon nanotubes (RVC-CNT) on reticulated glassy carbon. They found that RVC-CNT significantly promoted the electrical conductivity and electron-transfer rate of the biocathode, providing more reaction sites for the reduction of Cr(VI). The maximum power density of the modified MFC was 132.1 ± 2.8 mW/m^2^, and the removal rate attained 80.9%, which was 36.5% higher than that of the unmodified control group. Graphite felt biocathodes, modified using acid-pretreated, oxidized multi-walled carbon nanotubes, increased the removal rate of Cr(VI) from 0.97 ± 0.02 mg/(L·h) for the unmodified biocathodes to 2.00 ± 0.10 mg/(L·h) (Wu et al., 2017). Wu et al. (2016) constructed a biocathode using an NaX-type zeolite-modified graphite mat, whereby the internal resistance of the modified MFC was 162.30 Ω, compared to 337.01 Ω without modification. This corresponded to an increase in the maximum power density from 10.91 to 28.90 mW/m^2^, with the removal rate of Cr(VI) increasing to 10.39 ± 0.28 mg/(L·h), which was 8.2 times higher than that of the unmodified MFC. Song et al. (2016) used a graphene/biofilm composite to construct a biocathode with a maximum power density of 163.8 mW/m^2^, and the removal efficiency of Cr(VI) attained 100% after 48 h, which was significantly higher than the 17.5% of the graphite felt group. This was achieved because the graphene/biofilm-type biocathode could promote the transfer of electrons from the cathode to Cr(VI), thereby reducing Cr(VI). Although graphene has a higher specific surface area, the primary removal mechanism of Cr(VI) is biocatalytic reduction, rather than simple adsorption. Adding Sm-doped CeO_2_ nanoparticles to the carbon-based biocathode boosted its electrical performance from 31 to 113 mWh/m^2^. CeO_2_ nanoparticles are favored by the low redox potential between Ce^3+^ and Ce^4+^, which increases the active specific surface area of the cathode and can act as oxygen storage sites for bacteria (Marzorati et al., 2019). The high mobility of oxygen vacancies in the region enhances the electrocatalytic activity of the microorganisms; therefore, Sm-doping can increase the specific surface area of the biocathode, which can further enhance the power generation capacity of the MFC. A conventional carbon material was modified to create an abundant porous structure, which increased the specific surface area while supporting the effective adsorption of electroactive microorganisms. Overall, the internal resistance was reduced, and the electron-transfer rate was increased, thereby improving the removal efficiency of heavy metals by the biocathode.

### 3.2. Initial concentration and species of heavy metals

Heavy metal ions can act as electron acceptors in the cathode, and when the initial concentration of the heavy metal ions changes, the number of electron acceptors in the cathode also changes, thus affecting the cathodic half-reaction of the MFC. Huang et al. (2010) found that the initial concentration of Cr(VI) was positively correlated to the maximum power density of the MFC. An increase in the initial concentration of Cr(VI) from 12.8 to 39.2 mg/L corresponded to a decrease in the MFC coulomb efficiency from 98% ± 3 to 71% ± 2% and increase in the removal rate from 2.0 ± 0.1 mg/(g VSS·h) to 2.4 ± 0.2 mg/(g VSS·h). This result was considerably higher than the 0.14 mg/(L·h) removal rate of the abiotic cathode. Increasing the concentrations of Ni and Cd from 10 to 25 mg/L resulted in a decrease in removal from 91.7 and 86.9% to 48 and 33%, respectively, as higher heavy metal concentrations exceeded the tolerance of the microorganisms and inhibited the performance of the biocathode (Singh and Kaushik, 2021). Lin et al. (2021) determined that with an increase in the Cu(II) concentration, the microbial activity on the biocathode was inhibited, and the output voltage of the MFC rapidly decreased, followed by a gradual recovery of the output voltage as the Cu(II) concentration decreased. Increasing the initial concentration of heavy metals has been demonstrated to improve the power generation performance of MFCs and the removal effect of heavy metals without affecting the microbial activity of the biocathode.

When two or more heavy metals are simultaneously present, competition occurs between electron acceptors. For instance, when Cr(VI) and V(V) are simultaneously present in the cathode of an MFC, Cr(VI) is reduced first because the redox potential of Cr(VI) is higher than that of V(V). As the concentration of Cr(VI) gradually decreases and precipitates of Cr(III) form, the redox potential of V(V) eventually exceeds that of Cr(VI), and V(V) accepts electrons and is reduced. When V(V) is completely consumed, Cr(VI) becomes an electron acceptor again and continues to be reduced. In MFCs, both Cr(VI) and V(V) can be reduced, and the competition between them continues until one side is completely consumed. When both Cr(VI) and Cu(II) are present in the MFC cathode, Cu(II) can act as an electron mediator, thus reducing the diffusion internal resistance and cathodic overpotential of the MFC and promoting the power generation performance of the MFC and the reduction removal of Cr(VI). An increase in the concentration of Cu(II) from 10 to 50 mg/L raised the reduction rate of Cr(VI) from 1.080 to 1.191 g/(m^3^·h) (Li and Zhou, 2019). Wang et al. (2017) found that Fe(III) can reduce the internal diffusion resistance of Cr(VI) at the cathode and its reduction overpotential, because it acts as an electron mediator to obtain electrons from the cathode and transfers them to the reduced Cr(VI), rather than directly obtaining them from the cathode to achieve reduction. Notably, when several heavy metals are simultaneously present, the sequence or even competition or cooperation of heavy metal reduction needs to be clarified. In the MFC cathode, when oxygen and heavy metals simultaneously act as electron acceptors, the competition between them and the influence and mechanisms involved in the change of heavy metal morphology and cathodic reduction rate require further explanation.

### 3.3. pH

At the anode in the MFC, the organic matters produce protons and electrons. At the cathode, oxygen gains electrons and reacts with protons to form water or OH^−^ (O_2_ + 4e^−^ + 4H^+^ → 2H_2_O, O_2_ + 2H_2_O + 4e^−^ → 4OH^−^) (De Schamphelaire et al., 2008; Wang et al., 2016). Moreover, the rate of proton transfer to the cathode is lower than the rates of proton production at the anode and consumption at the cathode, resulting in a significant change in pH (Gil et al., 2003). Therefore, the pH of the cathode compartment increased and that of the anode compartment decreased. In addition, owing to the limitation of proton migration, the presence of an electric field can cause anodic acidification and cathodic alkalinization (Gil et al., 2003; Cang et al., 2012). Huang et al. (2015a) found that the optimal removal of Co(II) was achieved at pH 5.6, with a Co(OH)_2_ yield of 0.14 ± 0.01 mol/mol COD, which decreased to 0.09 ± 0.01 mol/mol COD at pH 6.1. Based on the potential–pH diagram, the potential of Cr(VI) increased with increasing H^+^ concentration; thus, the cathodic acidic environment is more favorable for Cr(VI) than for Cr(III), with the highest removal rate of Cr(VI) (96.47%) occurring at pH = 5. With a decrease in the pH to 3 and 4, the removal rate of Cr(VI) did not increase, which may be because the H^+^ in the cathode diffused to the anode, thus lowering the anode pH and inhibiting the activity of anode microorganisms (Zhao et al., 2021). For the sulfate-reducing bacterial biocathode, when Sb(V) and sulfate were simultaneously reduced, Sb precipitated as metal sulfide (Sb_2_S_3_) at the cathode, lowering the cathode pH from 7.0 to 5.2, and the removal rate of Sb(V) attained 99.28% (Arulmani et al., 2022). When both Cu(II) and Cr(VI) were present in the cathode, the pH of the solution had a significant effect on the reduction of the two heavy metals. At pH > 4, Cu(II) and Cr(VI) could be reduced simultaneously, and the reduction efficiency and power density of Cr(VI) decreased from 63 to 18% and from 4.4 to 1.1 mA/m^2^, respectively, with an increase in the concentration of Cu(II) from 50 to 500 mg/L (Gangadharan and Nambi, 2020). As abovementioned, the cathode pH of the MFC increased as the reaction proceeded, and numerous heavy metals produced various types of precipitates following the cathode reduction, which reduced the cathode activity. Therefore, maintaining the cathode pH in an acidic environment can enhance the effectiveness of the MFC, thereby offering significant advances in treating acidic heavy-metal wastewater.

### 3.4. Electrode potential

The redox potential required for the cathodic reduction of different heavy metals in MFCs varies widely, and heavy metals with higher redox potentials (e.g., Cr(VI) and Cu(II)) can be directly reduced as electron acceptors in MFC cathodes (Liang et al., 2009). Current was generated immediately when the biocathode potential was controlled at +50 and +150 mV, and the maximum power density (313 mA/m^2^) was obtained at +150 mV. Notably, no current was generated for 15 d at a cathode potential of +250 mV (Ter Heijne et al., 2010). Huang et al. (2011) set four different biological cathode potentials for Cr(VI) treatment. At cathode potentials of –150 and–300 mV, which provide the best metabolic energy for the microorganisms, the electroactive microorganisms on the cathode grew the quickest, and the current and Cr(VI) removal rates of the MFC were the highest. The reduction of Cr(III) was primarily present as precipitated Cr(OH)_3_ on the cathode, while only approximately 4% Cr(VI) was removed via bioadsorption under an open circuit condition. However, when the cathode redox potential did not match the redox potential required for heavy metal reduction, particularly for low-potential heavy metals [e.g., Cd(II), Co(II), and Pb(II)], an external power supply was required to control the cathode potential to achieve the thermodynamic reaction process of heavy metals. Shen et al. (2015) used MFC-driven, biocathode-type microbial electrolytic cells (MECs) to simultaneously remove Cu(II) (MFC) and Co(II) (MEC) without any external energy consumption. Zhang et al. (2015) constructed a gradient bioelectrochemical system for the progressive reduction removal of Cr(VI) and Cu(II) in a biocathode MFC, using the redox potentials of different heavy metals. This was followed by the removal of Cd(II) using the MEC biocathode (cathode potential of-530 mV). The presence of microorganisms reduced the energy required for electron transfer in the reduction reactions of these heavy metals (Qiu et al., 2017). Therefore, setting an appropriate cathode potential not only enhanced the treatment of heavy metal wastewater but also improved subsequent electricity generation.

### 3.5. Dissolved oxygen (DO)

Oxygen is the most popular terminal electron acceptor. When substrate degradation in the MFC anode is not a limiting factor, the oxygen concentration and reduction reaction in the biocathode directly determine the electrical production performance of the MFC (Ter Heijne et al., 2010). Moreover, oxygen and charge-transfer rates determine the performance of aerobic biocathodes (Milner and Yu, 2018). Rago et al. (2017) determined that *Spirulina* increased the DO in the biocathode and the thickness of the biofilm. In addition, ˃50% of the microbial species present were aerobic or microaerobic, such as *Halomonas* and *Pseudomonas,* which are able to enhance the redox reaction of cathodic O_2_, thereby improving the performance of the MFC (Rago et al., 2017). However, Huang et al. (2015a) found that a higher DO (0.222 mM) content decreased the recovery rate of Co(II) and the production of Co(OH)_2_ but favored the coulomb efficiency of the MFC and cathode. Ter Heijne et al. (2010) determined that the cathode potential in the presence of oxygen was significantly higher than that under anaerobic conditions (0.05–0.10 V), and the maximum output power density of the MFC was almost equal to twice that under anaerobic conditions, with Cu(II) removal efficiencies of 99.95 and 99.88%. Although the presence of oxygen enables competition with Cu(II) for electrons to inhibit the reduction of Cu(II), the nearly doubled enhancement of the MFC electrical production performance simultaneously promoted Cu(II) reduction; therefore, no significant difference in Cu(II) removal efficiency between the two conditions was observed. Liu et al. (2011) filled the cathode with air, which can significantly increase the reduction rate of Cr(VI). The iron-reducing bacteria on the biological cathode can oxidize O_2_ to H_2_O_2_, which can further promote the reduction of Cr(VI) to Cr(III). To enhance the dissolved oxygen in the cathode, researchers introduced photosynthetic microalgae into the cathode, which can fix CO_2_ and produce biomass, while releasing O_2_ into the cathode, thus enhancing the electricity production performance of the MFC (Yadav et al., 2020). Zhang et al. (2018) constructed a photosynthetic algae biocathode, generating a maximum output voltage of 266.1 mV, which was twice as high as that when ordinary nickel foam was used as the electrode. The Cd(II) removal rate attained ˃ 95%, and the polar functional groups of algae, including carbon, oxygen, and hydroxyl groups, were involved in cadmium adsorption (Zhang et al., 2018).

### 3.6. External resistors

When the external resistance is low, electrons are more likely to pass through the circuit and oxidize the substrate in the anode, whereby the efficient substrate oxidation rate is accompanied by a high electron carrier oxidation rate. Hence, the electrode reaction rate and mass-and charge-transfer processes are inhibited, while the output voltage of the MFC is lower and the current is higher (Jang et al., 2004; Menicucci et al., 2006). As the external resistance increases, the ohmic internal and charge-transfer resistances of the MFC increase, and the electron enrichment at the cathode increases the cathodic overpotential, which limits the electron migration to the cathode and the reduction of heavy metals at the cathode. Therefore, the reduction rate of Cr(VI) is faster when the external resistance ranges from 100 to 4,000 Ω (Li and Zhou, 2019). Wang et al. (2020) suggested that a lower external resistance would correspond to a higher MFC output current and more electrons supplied to the cathode, which can participate in the reduction of Cu(II). The migration of Cu(II) in the MFC requires a higher output voltage (i.e., larger external resistance) to achieve a higher migration or enrichment. Tao et al. (2011) found that the time required to achieve a cathodic Cu(II) removal rate ˃ 99% increased with increasing external resistance, because the MFC generates a higher current under low-resistance conditions in which electrons can pass to the cathode at a higher rate and increase the cathodic efficiency. Polarization curves were used to determine the current density and external resistance for power generation. Minimizing or even eliminating the external resistance can increase the electrical current and reduce metals. In the experimental data, the negative impact of high resistance was not always reflected in the removal efficiency but rather in the time taken to remove the metals.

## 4. Microbial community characteristics in biocathodes

Microorganisms are an important component of biocathodes, and the composition of the microbial community and abundance of the dominant species determine the effectiveness of the biocathode MFC in treating heavy metal wastewater (Table 1). Tandukar et al. (2009) established a biocathode two-chamber MFC and concluded that most of the Cr(VI) reduction was achieved via cathodic microorganisms. *Trichococcus pasteurii* and *Pseudomonas aeruginosa* were found to be involved in the reduction of Cr(VI) using 16 s RNA analysis. Current research on Cr(VI) removal using biocathodes is the most prevalent, with Cr(VI)-reducing microorganisms generally belonging to Proteobacteria, such as *S. oneidensis*, *Pseudomonas dechromaticans*, *Aeromonas dechromatica*, *Enterobacter coloacae*, *Desulfovibrio vulgaris*, and *Escherichia coli* (Hidayat et al., 2022). Cr(VI)-reducing microorganisms can also be isolated from several environments, such as soil, rivers, and anaerobic-activated sludge, including *Corynebacterium vitaeruminis* LZU47-1 (Zhao et al., 2021) and *Pseudomonas stutzeri* (Wu et al., 2015).

**Table 1:** Typical microbial species on the biocathode.

Heavy metal types	MFC configurations	Operating conditions (external resistance and initial concentration)	Cathode electrode material	Electrical production performance	Microorganisms	Removal rate/removal efficiency	Literature
Cr(VI)	Dual-chamber	1,000 Ω; 22–63 mg/L	Graphite sheet	55.5 mW/m^2^	*Trichococcus pasteurii*, *Pseudomonasaeruginosa*	0.46 mg Cr(VI)/(g VSS h)	Tandukar et al. (2009)
Cr(VI)	Dual-chamber	40 mg/L	Carbon felt	252.36 mW/m^2^	*Corynebacterium vitaeruminis* LZU47-1	98.63%	Zhao et al. (2021)
Cr(VI)	Dual-chamber	1,000 Ω; 20 mg/L	Graphite felt	9.7 mW/m^2^	*Gamma-proteobacteria*, *Pseudomonas stutzeri*	79.3% 0.66 ± 0.01 mg/(L h)	Wu et al. (2015)
Cr(VI)	Dual-chamber	1,000 Ω; 40 mg/L	Graphite felt	31.80 mW/m^2^	*Bacillus cereus*	60%, 2.56 ± 0.10 mg/(L h)	Wu et al. (2018)
Cr(VI)	Dual-chamber	1,000 Ω; 20 mg/L	HNO_3_-NaX zeolite-modified graphite felt	200 mW/m^2^	Mixed culture	10.39 mg/(L h)	Wu et al. (2016)
Cu(II)	Dual-chamber	510 Ω; 20 mg/L	Graphite brush	–	*Stenotrophomonas maltophilia* JY1, *Citrobacter* sp. JY3, *Pseudomonas aeruginosa* JY5, *Stenotrophomonas* sp. JY6	2.90 mg/(L h) 3.48 mg/(L h) 3.61 mg/(L h) 3.64 mg/(L h)	Tao et al. (2017)
Cu(II)	Single chamber	1,000 Ω; 172 mg/kg	Stainless steel mesh	34.9 mW/m^3^	*Proteobacteria*. *Firmicutes*, *Actinobacteria*	57%	Liu et al. (2021)
Cu(II)	Dual-chamber	1,000 Ω; 50–200 mg/L	Carbon cloth	41.3 mW/m^2^	*Trametes hirsute*, *Ganoderma lucidum*. *P. eryngii*	99%	Lin et al. (2021)
Cr(VI), Cu(II), Cd(II)	Dual-chamber MFC_Cr_-MFC_Cu_-MEC_Cd_	510 Ω; 5 mg/L	Graphite felt	48 μW (MFC_Cr_) 27 μW (MFC_Cu_) 994 μW (MEC_Cd_)	*Dysgonomonas, Azoarcus* (MFC_Cr_); *Geobacter, Myroides* (MFC_Cu_); *Achromobacter, Brucella, Alcaligenes, Tissierella, Brevundimonas* (MEC_Cd_)	1.21 ± 0.02 mg/(L h) (Cr) 1.18 ± 0.02 mg/(L h) (Cu) 1.15 ± 0.01 mg/(L h) (Cd)	Huang et al. (2015b)
Cu(II), Co(II)	MFCCu-MECCo (dual chamber)	Cu5-1,000 mg/L, Co40 mg/L	Porous graphite felt	27 W/m^3^	*Proteobacteria*	115.7 mg Cu/(L h). 6.4 mg Co/(L h)	Shen et al. (2015)
Sb(V)	Dual-chamber	1,000 Ω; 25 mg/L	Carbon felt	1652.9 W/m^3^	*Citrobacter freundii* SR10	99.28%	Arulmani et al. (2022)
Cd(II), Ni(II)	Dual-chamber	200 Ω; 10, 25 mg/L	Graphite brush	722 mW/m^3^	*Ochrobactrum*, *Halomonas*, *Achromobacter*	92% Ni, 87% Cd	Singh and Kaushik, (2021)
V(V)	Dual-chamber	100 Ω; 200 mg/L	Carbon felt	529 mW/m^2^	*Dysgonomonas*	>99%	Qiu et al. (2017)
U(VI)	Dual-chamber	1,000 Ω; 200 μM	Graphite felt	2.91 W/m^3^	*Pseudomonas*	90%	Vijay et al. (2020)

Sulfate-reducing bacteria are an efficient class of allotrophic metal-reducing bacteria that use sulfate as the final electron acceptor to produce sulfides. Arulmani et al. (2022) isolated a strain of sulfate-reducing bacteria (*Citrobacter freundii* SR10) from acid mine wastewater and constructed a biocathode that could remove both Sb(V) and $SO_{4}^{2-}$ . Singh and Kaushik (2021) used wetland sediments to culture biocathodes for the treatment of heavy metal wastewater containing Cd and Ni and found that the relative abundance of Proteobacteria in the biocathodes was 88.70%. Among these, *Ochrobactrum*, *Halomonas*, and *Achromobacter* were associated with Cd and Ni removal. Liu et al. (2021) constructed biocathodes by inserting a stainless-steel wire mesh into the sediment. After 100 days, Proteobacteria, Firmicutes, and Actinobacteria were the dominant clades in the biocathodes. Qiu et al. (2017) found that electroactive microorganisms on a biocathode could promote electron transfer and reduce the internal resistance of charge transfer. *Dysgonomonas* generated V(V) in the cathodic reduction, and the biocathode significantly improved the removal of V(V) under the synergistic effect of electrochemistry and microorganisms.

Despite the heterotrophic and phototrophic microorganisms with heavy metal reduction capability, electrotrophs are a fascinating group of microorganisms that harvest their energy by directly taking up electrons from the cathode and subsequently drive their metabolic processes coupled with heavy metal reduction. The critical function of electrotrophs was conferred by their inherent bioelectro-catalytical enzymes to facilitate the kinetic reaction of electron transfer, particularly under challenging neutral pH conditions. For instance, compared with the biocathode, 7–15% of the overall reduction rate of Cr(VI) was obtained in MFC abiotic cathodes, where the micro-niche of chromium-reducing microorganisms mitigate the shortage of the protons for Cr(VI) reduction and benefit the electron transport to the Cr(VI) (Tandukar et al., 2009). Shen et al. (2017) isolated the electro-trophic strain *Stenotrophomonas* sp. JY6 from Cu(II)-reduced biocathodes and tracked the Cu(II) subcellular distribution using a rhodamine-based fluorescent probe. Cathodic electrons mediated quicker Cu(II) entrance into the electrotrophic cytoplasm, accompanied with a 40% increase in Cu(II) removal rate. In conclusion, electrotrophs with heavy metal-reducing abilities represent a promising avenue for addressing environmental pollution and resource recovery challenges. Their capacity to use electrons from electrodes to detoxify and immobilize heavy metals provides innovative possibilities for sustainable bioremediation and the efficient reclamation of valuable metals from waste streams.

## 5. Conclusion

Biocathodes can reduce internal resistance and improve electron transfer. Therefore, biocathodes not only improve the electrical production performance of the MFC, but quickly remove heavy metals. Several key factors, such as electrode materials, initial heavy metal concentration, and pH, could affect the performance of the removal of heavy metals using biocathode MFCs. For different heavy metals, the corresponding dominant populations can be particularly cultivated on the cathode material to improve their removal rate. This can facilitate further investigation of the effect of different electrode materials in the treatment of heavy metal pollutants by the biocathode MFC, as well as the selection of the electrode materials that are more favorable for electrons. In addition, existing studies on the treatment of heavy metal wastewater using biocathode MFCs have primarily focused on single heavy metal ions, and further study on the treatment of multiple, mixed heavy metals using biocathodes should be performed. Although the removal of heavy metal pollutants using biocathode MFCs is still far from a practical application, this mechanism will be further improved as research progresses.

## Author contributions

HW: Conceptualization, Project administration, Writing – original draft. PZ: Methodology, Writing – original draft. XL: Supervision, Writing – review & editing. JM: Data curation, Writing – original draft. YL: Writing – review & editing, Investigation, Methodology. BL: Resources, Writing – original draft. ZX: Funding acquisition, Supervision, Writing – review & editing.

## Funding

The author(s) declare financial support was received for the research, authorship, and/or publication of this article. This research was funded by the National Natural Science Foundation of China (42107030 and 42207073), Natural Science Foundation of Hunan Province [2023JJ40539].

## Conflict of interest

The authors declare that the research was conducted in the absence of any commercial or financial relationships that could be construed as a potential conflict of interest.

## Publisher’s note

All claims expressed in this article are solely those of the authors and do not necessarily represent those of their affiliated organizations, or those of the publisher, the editors and the reviewers. Any product that may be evaluated in this article, or claim that may be made by its manufacturer, is not guaranteed or endorsed by the publisher.
